# Hepatitis C Viremia Is Associated with Cytomegalovirus IgG Antibody Levels in HIV-Infected Women

**DOI:** 10.1371/journal.pone.0061973

**Published:** 2013-04-17

**Authors:** Mark H. Kuniholm, Christina M. Parrinello, Kathryn Anastos, Michael Augenbraun, Michael Plankey, Marek Nowicki, Marion Peters, Elizabeth T. Golub, Nell Lurain, Alan L. Landay, Howard D. Strickler, Robert C. Kaplan

**Affiliations:** 1 Department of Epidemiology and Population Health, Albert Einstein College of Medicine, Bronx, New York, United States of America; 2 Department of Medicine, Montefiore Medical Center, Bronx, New York, United States of America; 3 Department of Medicine, State University of New York Downstate Medical Center, Brooklyn, New York, United States of America; 4 Department of Medicine, Georgetown University Medical Center, Washington, D. C., United States of America; 5 Department of Medicine, University of Southern California, Los Angeles, California, United States of America; 6 Department of Medicine, University of California San Francisco, San Francisco, California, United States of America; 7 Department of Epidemiology, Johns Hopkins Bloomberg School of Public Health, Baltimore, Maryland, United States of America; 8 Department of Immunology/Microbiology, Rush University Medical Center, Chicago, Illinois, United States of America; 9 Division of Pharmacology, Utrecht Institute of Pharmaceutical Sciences, Faculty of Science, Utrecht University, Utrecht, The Netherlands; Alberta Provincial Laboratory for Public Health/University of Alberta, Canada

## Abstract

**Background:**

Individuals with HIV infection exhibit high cytomegalovirus (CMV) IgG levels, but there are few data regarding the association of hepatitis C virus (HCV) with the immune response against CMV.

**Methods:**

Associations of HCV with CMV seropositivity and CMV IgG levels were studied in 635 HIV-infected women, 187 of whom were HCV-seropositive, with adjustment in multivariable models for age, race/ethnicity, and HIV disease characteristics. Eighty one percent of the women reported receipt of highly active antiretroviral therapy (HAART) prior to or at CMV testing.

**Results:**

In adjusted models women with chronic HCV had higher CMV IgG levels than those without HCV RNA (β = 2.86, 95% CI:0.89 – 4.83; *P* = 0.004). The association of HCV RNA with CMV IgG differed by age (*P*_interaction_ = 0.0007), with a strong association observed among women in the low and middle age tertiles (≤45.3 years of age; β = 6.21, 95% CI:3.30 – 9.11, *P*<0.0001) but not among women in the high age tertile. CMV IgG levels were not associated with non-invasive measures of liver disease, APRI and FIB-4, or with HCV RNA level and adjustment for Epstein-Barr virus (EBV) IgG levels did not affect the association between HCV and CMV.

**Conclusions:**

CMV IgG levels are higher in HCV/HIV co-infected women than in HIV mono-infected women. Further research on the association of HCV with CMV IgG is indicated because prior studies have found CMV IgG to be associated with morbidity and mortality in the general population and subclinical carotid artery disease in HIV-infected patients.

## Introduction

Cytomegalovirus (CMV) is a human β herpesvirus with tropism for a wide range of cell types that establishes a life-long, persistent infection [1]. Primary infection with CMV in immunocompetent individuals induces strong innate and adaptive immune responses which control viral replication and prevent CMV-associated morbidity and mortality [2]. Control of CMV replication, however, requires significant investment of immune resources and a large fraction of the circulating T-cell population is CMV-specific in CMV-infected individuals [3], [4]. Individuals with defects in immune function, such as the elderly and individuals with HIV infection, invest an even greater proportion of immune resources to control CMV viremia. Specifically, the CMV-specific T-cell population is significantly expanded in these populations [5], [6], and the elderly and individuals with HIV infection also show higher CMV IgG antibody levels as compared to younger and HIV-uninfected individuals, respectively [7]–[9].

Chronic infection with hepatitis C virus (HCV) leads to exhaustion and death of HCV-specific T-cells (reviewed in [10]) but may also cause defects in overall immune function. For example, peripheral blood dendritic and naïve CD4+ T-cells are reduced in number and function in individuals with chronic HCV [11], [12], and peripheral blood CD4+ T-cell levels are also reduced in individuals with HCV-associated cirrhosis, perhaps because CD4+ T-cells are sequestered in the tissues of these individuals [13]. Given these data, we hypothesized that chronic HCV infection may influence the immune response against CMV in a similar fashion to old age and HIV infection.

The association of HCV infection with CMV pathogenesis has been extensively studied in liver transplant patients (reviewed in [14]), and more recently in patients receiving HCV antiviral therapy [15]. We are aware of only one small study that examined the association of HCV infection with measures of the immune response against CMV [16]. The results of that study showed that both CMV seroprevalence and CMV IgG levels differed by HCV infection status [16]. An important limitation of that study, though, aside from its small sample size was that age and other potentially confounding factors were not accounted for in the analysis.

In the current study we examined the relationship of HCV infection with CMV seropositivity and IgG antibody levels in a large cross-sectional population of HIV-infected women with a high prevalence of HCV co-infection. First, we studied age- and HIV treatment group-specific associations and used multivariable models to account for potentially confounding effects of age, race/ethnicity, decade of participant recruitment, and HIV disease characteristics. Second, we added interaction terms to our fully adjusted multivariable models to assess if associations of HCV with CMV IgG levels were similar between strata of age and among HIV treatment groups. Third, we examined whether CMV IgG levels were associated with non-invasive measures of liver disease, APRI and FIB-4, and with HCV RNA level in women with chronic HCV infection. Fourth, we assessed whether HCV infection might also be associated with the humoral immune response against another herpesvirus, Epstein-Barr virus (EBV), by studying the relationship between HCV infection and EBV IgG levels in these same women. Lastly, we added EBV IgG to our multivariable models to evaluate the independence of associations of HCV and EBV with CMV.

## Materials and Methods

### Study population

The Women's Interagency HIV Study (WIHS) is a prospective, multicenter cohort study of 2,791 HIV-seropositive and 975 at-risk HIV-seronegative women enrolled through similar sources at six clinical sites. The initial enrollment was conducted during 1994 – 1995 and a second recruitment occurred during 2001 – 2002. WIHS women are followed semi-annually with physical exams, specimen collection including blood, and detailed questionnaires regarding health and behavior [17].

In April 2004 all WIHS women were invited to participate in a carotid artery disease substudy [18], and this substudy was completed by 75% of both HIV-infected and HIV-uninfected women. CMV and EBV IgG antibodies were measured in plasma collected in 2004 – 2005 in the first 650 HIV-infected women who were enrolled in the carotid artery substudy to investigate associations between these antibody levels and subclinical cardiovascular disease and other outcomes, as described previously [9]. All WIHS women were tested for HCV antibody upon enrollment, and most HCV antibody positive samples were tested for HCV RNA. Women who were tested for CMV IgG and were HCV antibody positive but lacked HCV RNA data [N = 10] were excluded from the current study, as were women with only a single HIV RNA measurement [N = 5], because these limited data precluded us from accurately characterizing the HIV viremia status of these women (see Statistical Methods). Two women who were HCV antibody positive and HCV RNA negative, and who were enrolled in 1994 – 1995, reported receipt of interferon alpha monotherapy prior to enrollment. It is unknown whether this interferon therapy was prescribed as treatment for chronic HCV infection, or to treat other conditions (e.g, AIDS- related Kaposi's sarcoma).

### Ethics Statement

Ethics approval for this study was provided by the institutional review board of the Albert Einstein College of Medicine. The WIHS protocol was approved by each local institutional review board, and all participants provided written informed consent.

### Laboratory testing

#### CMV Testing

CMV IgG antibody levels were determined using the CMV ELISA Quantitation Kit (GenWay Biotech, Inc., San Diego, CA). A 2.5 µL volume of each serum sample was diluted 1∶80 to obtain samples within the readable range of the assay. Binding of CMV-specific IgG antibodies to the antigen-coated wells was detected by horse radish peroxidase-conjugated goat anti-human IgG followed by addition of enzyme substrate 3,3′,5,5′-tetramethybenzidine (TMB). Manufacturer-provided positive and negative controls were included on each plate, and the optical density (OD) of the colored product was read at 450 nm using a DuPont Kinetic Microplate Reader (Molecular Devices, Sunnyvale, CA). CMV seropositivity was defined by the manufacturer as >1.1 IU/mL and we used this cut-off for the current study because this assay format has undergone extensive validation [19], [20]. Kits from the same lot number were used for all of the samples. The intra-assay coefficient of variation (CV) for CMV IgG levels ranged from 2.4%–8.0% and the inter-assay CV ranged from 5.2%–9.9%.

#### EBV Testing

EBV IgG antibody levels were determined using the EBV VCA ELISA Kit (GenWay Biotech, Inc., San Diego, CA). A 5 µL volume of each serum sample was diluted 1∶100. The procedure for detection of EBV-specific IgG antibodies was the same as that described above for CMV IgG, and EBV seropositivity was defined as suggested by the manufacturer. The intra-assay CV was 9.4% and the inter-assay CV ranged from 1.6–14.0%.

#### HCV Testing

HCV serostatus was determined at enrollment using a commercial second- or third-generation enzyme immunoassay, and HCV viremia was determined in HCV-seropositive women using either the COBAS Amplicor Monitor 2.0, which has a linear range of 600–5.0 × 10^5^ IU/ml, as previously described [21], or the COBAS Taqman assay, which has a linear range of 10–2.0 × 10^8^ IU/ml (both from Roche Diagnostics, Branchburg, NJ). Follow-up HCV RNA testing was conducted on most but not all women who were HCV RNA positive at enrollment using the Amplicor or Taqman assay.

#### HIV Testing

Plasma HIV RNA levels were assessed by PCR with assays that have lower levels of detection of 80 copies/mL and CD4+ T-cell counts were determined by flow cytometry in laboratories participating in the DAIDS Quality Assurance Program.

#### Liver Disease Assays

Aspartate and alanine aminotransferase (AST and ALT) levels and platelet counts were determined using standard laboratory protocols at the six clinical sites. We calculated the APRI index (100*[AST/AST ULN]/platelet count [10^9^/L]) and the FIB-4 index ([Age*AST]/[platelet count (10^9^/L)*ALT^½^]) as described previously [22], [23]. In keeping with prior papers [22], [23], an APRI≤0.5 was defined as the threshold indicating no significant liver fibrosis, whereas an APRI>1.5 was considered evidence of significant fibrosis. For FIB-4 these thresholds were <1.45 and >3.25, respectively.

### Statistical methods

Initial analyses examined differences in clinical characteristics by HIV treatment status - we defined women as: a) untreated – if they reported never having received highly active antiretroviral therapy (HAART), b) treated and aviremic – if they reported receipt of HAART at or prior to the visit at which CMV was measured and had HIV RNA levels at or below the lower level of detection for ≥50% of study visits following first reported HAART, and c) treated and viremic – if they reported receipt of HAART and had HIV RNA levels above the lower level of detection for >50% of visits following first reported HAART. Tenofovir, Lamivudine and FTC Emtriva were the most commonly used antiretroviral therapies at the CMV testing visit, used by 273, 235and 186 women, respectively.

To facilitate interpretation, we defined HCV infection status as chronic HCV (HCVAb+/HCV RNA+), cleared HCV (HCVAb+/HCV RNA-), or HCV-uninfected (HCVAb-) based on testing of plasma collected at WIHS enrollment. Women with chronic HCV were assumed to be HCV RNA positive while other women were assumed to be HCV RNA negative, with the acknowledgement that there could be some misclassification of HCV RNA status at the time of CMV and EBV testing in 2004–2005. Specifically, some women [N = 11] were tested for HCV RNA only once at enrollment while other women [N = 9] were HCV RNA positive at enrollment but HCV RNA negative during follow-up HCV RNA testing in 2006–2007 (see Discussion).

We examined the cross-sectional relationship of HCV infection status with CMV seropositivity using Fisher's exact tests. Then, among CMV-seropositives, we studied CMV IgG levels in relation to HCV infection status. Because CMV IgG levels did not differ significantly between women with cleared HCV as compared to HCV-uninfected women, we combined the cleared HCV and HCV-uninfected groups. All subsequent analyses compared those women who were HCV RNA positive to those who were HCV RNA negative.

The impact of age and HIV treatment group on associations of HCV RNA status with CMV IgG levels in CMV-seropositives was assessed by calculating age- and HIV treatment group-specific median CMV IgG levels. We then studied associations of HCV RNA status with CMV IgG levels using multivariable linear regression models because CMV IgG levels were distributed approximately normally (data not shown). In these models, we adjusted for age (in tertiles), race/ethnicity, decade of participant recruitment, HIV treatment group (defined above), current CD4+ T cell count (<200, 200 – 500, >500 cells/µL), and nadir CD4+ T cell count (<200, 200 – 500, >500 cells/µL). Tests for statistical interaction were conducted by including product terms in the fully adjusted models. Four women with missing CD4+ T-cell count data at their CMV testing visit were excluded from these multivariable analyses.

To study the association between non-invasive measures of liver disease and CMV IgG levels we limited our analyses to women with chronic HCV infection and to those without missing concurrent AST, ALT and platelet measurements. Linear regression models with adjustments as described above were used to study APRI and FIB-4 in relation to CMV IgG levels. APRI and FIB-4 were parameterized both as continuous variables and as three categories – levels consistent with fibrosis, indeterminate levels, and levels consistent with no fibrosis (see Laboratory Methods).

The association between CMV IgG and HCV RNA levels was also examined in the subset of women with chronic HCV infection. For this analysis, we used HCV RNA levels measured at the enrollment visit because very few of the studied women were tested for HCV RNA and CMV IgG concurrently at the same study visit. We calculated Pearson's correlation coefficient to assess the relationship between enrollment log_10_ HCV RNA and CMV IgG levels. We then studied the association between HCV RNA status and EBV IgG levels among women who were EBV-seropositive. These analyses were also conducted using multivariable linear regression models – because EBV IgG was distributed approximately normally (data not shown) - with adjustment for the factors listed above. Lastly, we included EBV IgG as a covariate in the fully adjusted model of HCV RNA with CMV IgG to assess the independence of associations of HCV and EBV with CMV.

## Results

### Demographic and clinical characteristics of the study population

Selected characteristics of the 635 HIV-infected women in this study are shown in Table 1. Study women were largely in their late thirties to early forties at the time of CMV testing and were majority Black, non-Hispanic. Eighty one percent of the women reported receipt of HAART prior to or at CMV testing in 2004 – 2005. Among HAART-treated women, 42% had undetectable HIV RNA levels at the majority of their post-HAART study visits (designated the aviremic HIV subgroup). As expected, HAART-treated women who were aviremic had higher current CD4+ T-cell levels than both HAART-treated viremic and untreated women (medians: 581, 314, 495 cells/µL; interquartile ranges (IQRs): 443 – 728, 213 – 489, 345 – 668 for aviremic, viremic, and untreated women, respectively: *P*<0.001 for both comparisons). Women who were aviremic also had higher nadir CD4+ T-cell levels as compared to viremic women (medians: 244, 181 cells/µL; IQRs: 122 – 358, 90 – 290, for aviremic and viremic women, respectively: *P* = 0.002), but nadir CD4+ T-cell level was lower for both groups of HAART-treated women compared to untreated women (median in untreated women: 401 cells/µL; IQR: 291 – 549: *P*<0.001 for both comparisons).

**Table 1:** Characteristics of the study women (N = 635).

Age, median (IQR)	41 (36–47)
Race/Ethnicity	
Black, non-Hispanic	396 (62%)
White, non-Hispanic	57 (9%)
Hispanic	171 (27%)
Other	11 (2%)
Recruitment cycle	
1994–95	382 (60%)
2001–02	253 (40%)
HCV status^a^	
HCV antibody-	448 (70%)
HCV antibody+/HCV RNA-	43 (7%)
HCV antibody+/HCV RNA+	144 (23%)
Current CD4+ T-cell count (cells/µL)^b^	
<200	81 (13%)
200–500	285 (45%)
>500	265 (42%)
Nadir CD4+ T-cell count (cells/µL)	
<200	260 (41%)
200–500	297 (47%)
>500	78 (12%)
HIV subgroup^c^	
Untreated	122 (19%)
Receipt of HAART and aviremic	218 (34%)
Receipt of HAART and viremic	295 (47%)

IQR, interquartile range; HAART, highly active antiretroviral therapy.

a HCV status was determined at enrollment. Follow-up HCV RNA testing was conducted on all women who were HCVAb+ at enrollment and HCV RNA status was 100% concordant between enrollment and follow-up testing (see Laboratory Methods).

b Four women had missing CD4+ data at their CMV testing visit.

c Untreated: no receipt of HAART; Treated and aviremic: undetectable HIV RNA for ≥50% of study visits following first reported receipt of HAART; Treated and viremic: HIV RNA levels above the lower level of detection for >50% of study visits following first reported receipt of HAART.

### CMV seropositivity by HCV infection status

Among the 635 study women, 594 (94%) were CMV seropositive. The prevalence of CMV seropositivity did not differ significantly by HCV infection status - seropositivity was 93%, 93%, and 94%, among those with chronic HCV, cleared HCV, and in HCV-uninfected women, respectively (*P* = 0.89).

### CMV IgG antibody levels by HCV infection status

Among CMV-seropositives, those with chronic HCV infection had higher CMV IgG levels (median: 27.5 IU/mL; IQR: 21.4 – 36.2) than those with cleared HCV (median: 26.2 IU/mL; IQR: 16.0 – 32.6) and HCV-uninfected women (median: 25.0 IU/mL; IQR: 16.2 – 31.4). Differences between women with chronic HCV vs. cleared HCV were marginally significant (*P* = 0.05), whereas differences between women with chronic HCV vs. HCV-uninfected women were highly significant (*P* = 0.0001). CMV IgG levels did not differ significantly between women with cleared HCV vs. HCV-uninfected women (*P* = 0.83).

**Figure 1:**
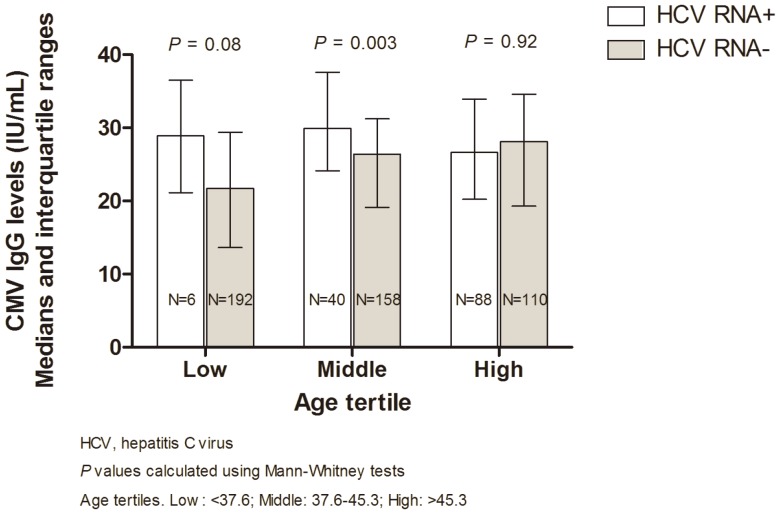
Median CMV IgG (IU/mL) by HCV RNA status and age tertile in CMV-seropositive women (N = 594).

Figure 1 and Table 2 show CMV IgG levels by HCV RNA status stratified by tertiles of age (Figure 1) and by tertiles of age and HIV treatment subgroup (Table 2). As shown in Figure 1, women in the lowest age tertile (younger than 37.6 years old) and middle age tertile (37.6 – 45.3 years old) with chronic HCV had higher CMV IgG levels than HCV RNA negative women (*P* = 0.08 and *P* = 0.003 for the lowest and middle age tertile comparisons, respectively). In contrast, CMV IgG levels in women in the highest age tertile (>45.3 years old) did not differ by HCV RNA status (*P* = 0.92).

**Table 2:** Median CMV IgG levels (IU/mL) stratified by HCV RNA status, age tertile, and HIV treatment status among CMV-seropositive women.

	HAART-treated/aviremic (N = 205)					HAART-treated/viremic (N = 273)					Untreated (N = 116)				
	HCV RNA+^a^		HCV RNA-			HCV RNA+^a^		HCV RNA-			HCV RNA+^a^		HCV RNA-		
	N	Median (IQR)	N	Median (IQR)	*P* b	N	Median (IQR)	N	Median (IQR)	*P* b	N	Median (IQR)	N	Median (IQR)	*P* b
Low and middle age tertilesc, d	14	34.0	125	21.0	0.003	22	33.6	161	27.1	0.002	10	23.4	64	22.1	0.39
		(21.1–38.1)		(14.3–28.9)			(26.7–37.6)		(19.9–32.0)			(21.6–28.1)		(11.5–28.4)	
High age tertile^c^	32	26.9	34	27.8	0.76	39	26.8	51	27.4	0.90	17	24.7	25	28.8	0.74
		(19.9–35.3)		(19.1–33.9)			(19.8–34.2)		(21.0–34.6)			(22.9–31.3)		(17.3–36.7)	

a Women who were HCVAb+/HCV RNA+ at enrollment were defined to be HCV RNA+ while those who were HCV Ab+/HCV RNA- and HCVAb- at enrollment were defined to be HCV RNA-. Follow-up HCV RNA testing was conducted on all women who were HCVAb+ at enrollment and HCV RNA status was 100% concordant between enrollment and follow-up testing. Follow-up testing of HCVAb- women was not conducted (see Laboratory Methods and Discussion).

b *P* values calculated using Mann-Whitney tests.

c Age tertiles. Low: <37.6; Middle: 37.6–45.3; High: >45.3. The low and middle age tertiles were combined because of the small number of HCV RNA positive women in the low age tertile.

As shown in Table 2, HAART-treated women in the lowest and middle age tertiles with chronic HCV had higher CMV IgG levels than comparably-aged, HAART-treated, HCV RNA negative women (*P* = 0.003 and *P* = 0.002 for the aviremic and viremic women, respectively). CMV IgG levels did not differ, though, by HCV RNA status among HAART-treated women in the highest age tertile or among untreated women.

In multivariable models that adjusted for age, race/ethnicity, decade of participant recruitment, HIV treatment group, current CD4+ T-cell level, and nadir CD4+ T-cell level, women with chronic HCV had higher CMV IgG levels than women without HCV RNA (β = 2.86, 95% CI:0.89 – 4.83; *P* = 0.004). In interaction models that included adjustment for the factors listed above, the association of HCV RNA with CMV IgG level differed significantly by age (low and middle tertiles vs. high age tertile: *P*_interaction_ = 0.0007) but not by HIV treatment status (HAART-treated vs. untreated: *P*_interaction_ = 0.57). Consistent with the significant interaction by age, associations of HCV RNA with CMV IgG level were highly significant in multivariable analyses restricted to 394 women in the low and middle age tertiles (β = 6.21, 95% CI: 3.30 – 9.11; *P*<0.0001) but not when restricted to 196 women in the high age tertile (β = 0.67, 95% CI: −2.09 – 3.48; *P* = 0.63).

### CMV IgG antibody levels by APRI and FIB-4 in HCV RNA positives

Among 129 women with chronic HCV and available AST, ALT and platelet measurements, 12% had APRI levels indicative of fibrosis, 41% had indeterminate levels, and 47% had APRI levels consistent with no fibrosis. For FIB-4 these percentages were 19%, 46%, and 35%. 128 of these women had complete data for multivariable analysis, and among these women APRI and FIB-4 levels were not associated with CMV IgG levels (β = 1.69, 95% CI: −0.39 – 3.77; *P* = 0.11 and β = 0.76, 95% CI: −0.36 – 1.89; *P* = 0.18, respectively) in models that adjusted for age, race/ethnicity, decade of recruitment, HIV treatment group, current CD4+ level, and nadir CD4+ level. When APRI and FIB-4 were treated as categorical variables (i.e., two indicator variables for APRI/FIB-4: i) levels consistent with fibrosis, ii) indeterminate levels - APRI/FIB-4 consistent with no fibrosis was the reference) the results in adjusted models were similarly null (β = 3.73, 95% CI: −1.45 – 8.91; *P* = 0.16 and β = −1.42, 95% CI: −4.91 – 2.07; *P* = 0.42 for APRI and β = 1.31, 95% CI: −3.60 – 6.21; *P* = 0.60 and β = −0.38, 95% CI: −4.12 – 3.36; *P* = 0.84 for FIB-4).

### CMV IgG antibody levels by HCV RNA level in HCV RNA positives

Among 144 women with chronic HCV infection, CMV IgG level was positively associated with log_10_ HCV RNA level measured at enrollment, but this association was not statistically significant (r = 0.13, *P* = 0.14).

### EBV IgG antibody levels by HCV RNA status

Among the 635 study women, 629 (99%) were EBV seropositive. Among EBV-seropositive women, HCV RNA status was not associated with level of EBV IgG (β =  −4.94, 95% CI: −17.23 – 7.36; *P* = 0.43) in models that adjusted for age, race/ethnicity, decade of participant recruitment, HIV treatment group, current CD4+ T-cell level, and nadir CD4+ T-cell level.

### Effect of EBV IgG on the association of HCV RNA with CMV IgG antibody levels

Independent associations of EBV IgG, HCV RNA, and other covariates with CMV IgG levels in 584 women who were both CMV and EBV seropositive and had available CD4+ T-cell count data are shown in Table 3. The association of HCV RNA status with CMV IgG was essentially unchanged following adjustment for EBV IgG in addition to the other covariates listed above (β = 3.08; 95% CI:1.16 – 5.00; *P* = 0.002 with adjustment for EBV IgG vs. β = 2.86, 95% CI:0.89 – 4.83; *P* = 0.004 without adjustment for EBV).

**Table 3:** Independent associations of HCV RNA status and covariates with CMV IgG (IU/mL)^a^.

	β	Lower 95% CI	Upper 95% CI	*P* value
HCV RNA+ (vs. HCV RNA-)^b^	3.08	1.16	5.00	0.002
EBV IgG (continuous)	0.04	0.02	0.05	<0.001
High age tertile (vs. low age tertile)^c^	3.46	1.25	5.67	0.002
Middle age tertile (vs. low age tertile)^c^	2.90	0.96	4.84	0.003
Hispanic (vs. Black)	−1.54	−3.27	0.20	0.08
Other (vs. Black)	3.55	−2.43	9.52	0.24
White (vs. Black)	−1.80	−4.66	1.06	0.22
2001–2002 recruit (vs. 1994–1995)	1.43	−0.33	3.18	0.11
Untreated (vs. treated and viremic)^c^	−2.53	−4.70	−0.36	0.02
Treated and aviremic (vs. treated and viremic)^d^	−2.26	−4.09	−0.42	0.02
Current CD4+ count 200–500 (vs. ≥500)	−0.16	−2.00	1.68	0.86
Current CD4+ count <200 (vs. ≥500)	−1.96	−4.88	0.97	0.19
Nadir CD4+ count 200–500 (vs. ≥500)	1.30	−1.38	3.98	0.34
Nadir CD4+ count <200 (vs. ≥500)	3.51	0.39	6.63	0.03

a Results from a single multivariable linear regression model of N = 584 women who were both CMV and EBV seropositive and had CD4+ T-cell data at their CMV testing visit.

b Women who were HCVAb+/HCV RNA+ at enrollment were defined to be HCV RNA+ while those who were HCV Ab+/HCV RNA- and HCVAb- at enrollment were defined to be HCV RNA-. Follow-up HCV RNA testing was conducted on all women who were HCVAb+ at enrollment and HCV RNA status was 100% concordant between enrollment and follow-up testing. Follow-up testing of HCVAb- women was not conducted (see Laboratory Methods and Discussion).

c Age tertiles. Low: <37.6; Middle: 37.6–45.3; High: >45.3.

d Untreated: no receipt of HAART; Treated and aviremic: undetectable HIV RNA for ≥50% of study visits following first reported receipt of HAART; Treated and viremic: HIV RNA levels above the lower level of detection for >50% of study visits following first reported receipt of HAART.

## Discussion

Our results show that HIV-infected women with chronic HCV have significantly higher CMV IgG levels than HIV-infected women without HCV RNA. Specifically, women with chronic HCV infection had, in adjusted models, CMV IgG levels that were 3.08 IU/mL higher than those of women without HCV RNA. Although the relationship between CMV IgG and CMV replication in tissues is not well understood, high CMV IgG levels are associated with increased incidence of subclinical atherosclerosis [24], coronary heart disease [25] and with cardiovascular and all-cause mortality [26]–[28] in the general population and also with subclinical carotid artery disease in HIV-infected individuals [9]. There is also growing evidence that the immune response against CMV contributes to immunosenescence and the pathogenesis of other diseases [29]. The association of chronic HCV infection with high CMV IgG levels is therefore interesting because it suggests a biologic mechanism through which HCV could contribute to the pathogenesis of a variety of chronic diseases. HCV contributes to liver disease and insulin resistance through well described pathways (reviewed in [30]), but to date it is unclear if and how HCV contributes to all-cause [31], [32] and cardiovascular mortality [32] through other mechanisms.

The association of chronic HCV with high CMV IgG levels was significant only among women younger than 46 years of age in our investigation. CMV IgG levels are on average higher in older as compared to younger individuals [8], [26] and are also elevated in HIV-positives [7], [9]. It is therefore possible that the CMV-specific immune response is maximally committed in older HIV-positive individuals, and that the additional impact of chronic HCV is negligible in these individuals. Other explanations for the observed interaction by age may also exist, but larger studies involving both HIV-infected and HIV-uninfected individuals with a wide range of ages will be needed to more completely characterize differences in HCV associations with CMV IgG by age.

In contrast to the results of a prior study [16], we found no evidence that CMV seropositivity differs by HCV infection status. CMV seropositivity was very common in the women studied in our investigation, consistent with data showing that CMV seroprevalence is high among racial/ethnic minorities and low-income individuals in the US [33], [34]. We also did not observe differences in the association of HCV with CMV IgG level by HIV treatment group in multivariable analysis, despite a suggestion that such a difference might exist in the unadjusted data (Table 2). We note, though, that 81% of our study population was HAART-treated and so populations with larger proportions of HAART-naïve participants may have greater power to examine this question.

CMV IgG levels were not associated with concurrently measured markers of liver disease, APRI and FIB-4, or with HCV RNA level at the enrollment visit in the women with chronic HCV infection. These data, therefore, do not provide support for the hypothesis that high CMV IgG levels accelerate the progression of liver disease in individuals with chronic HCV infection. It is possible, though, that CMV IgG levels are associated with progression of cardiovascular or other extrahepatic diseases in individuals with chronic HCV. Future studies that include large numbers of HCV-positive individuals will be needed to more completely characterize the pathogenesis of CMV in HCV-infected populations.

Chronic HCV infection was not associated with EBV IgG levels in HIV-infected WIHS women, nor did adjustment for EBV IgG affect the observed association between HCV and CMV IgG. These data suggest that chronic HCV infection may not be associated with the humoral immune response against other herpesviruses. Further studies in other populations will be required, though, to more fully address the specificity of the HCV-CMV relationship.

An important limitation of the current study is that it is cross-sectional. We are therefore unable to exclude the possibility that high CMV IgG levels influence the likelihood of HCV clearance, even though we believe that this possibility is less likely than an effect of chronic HCV infection on CMV IgG levels. It is also possible that the same host genetic factors influence both HCV clearance and CMV IgG levels. Genetic associations with HCV clearance have been extensively studied (reviewed in [35]), but we are not aware of any strong genetic associations with CMV IgG [36].

A second limitation is that there could be some misclassification of HCV status, as we defined chronic HCV infection based on the presence of HCV RNA at the enrollment visit. Eleven women who were HCV RNA positive at enrollment did not have follow-up HCV RNA testing and it is possible that some of these women cleared HCV RNA spontaneously during follow-up. Another 9 women were found to be HCV RNA negative after the date of CMV testing, meaning they cleared HCV RNA spontaneously sometime before or after CMV testing was conducted. We also note that follow-up HCV testing of women who were HCV-seronegative at WIHS enrollment has not been conducted. Injection drug use (IDU) declined dramatically among WIHS women following enrollment, but even if many WIHS women acquired new HCV infections after enrollment, or if a small number of women classified as having chronic HCV infection were actually HCV RNA negative at the CMV testing visit, the study results would still be biased toward rather than away from the null (i.e., increasing the probability of a type II rather than a type I error). Another limitation of this study is that we did not have data related to CMV DNA or CMV-specific T cell responses. A large study of HIV patients with low CD4+ T cell levels (median: 80 cells per µL) detected CMV DNA in the blood of only 16% of participants [37] suggesting that CMV DNA in the blood is unlikely to be a good biomarker of CMV pathogenesis in populations with less pronounced immune suppression. CMV DNA extracted from purified monocytes could prove to be a useful epidemiologic biomarker, though, because CMV DNA could be detected in 58% of community-dwelling older adults using this method [38]. Large studies that incorporate CMV IgG, CMV-specific T cell response and CMV DNA data are clearly needed to more completely understand the complex interactions between CMV, HCV and HIV and to relate these biomarkers to clinically important phenotypes.

Overall, our study indicates for the first time to our knowledge that individuals with HCV/HIV co-infection have higher CMV IgG antibody levels as compared to HIV mono-infected individuals. Further research on the association of HCV with CMV IgG is indicated because of the strong associations of high CMV IgG levels with morbidity and mortality. In particular, it is not known if our findings will replicate in HCV mono-infected populations, or in populations with a wide distribution of ages. As many as 5.2 million individuals in the US are living with chronic HCV [39], and the majority of these individuals are also likely to be infected with CMV. A better understanding of the interaction of these two chronic viral infections could lead to better management strategies for HCV-infected individuals.
